# Lectures on the Diseases of the Urinary Organs

**Published:** 1835-07-01

**Authors:** 


### MEDICAL QUARTERLY REVIEW.

### REVIEWS.

*Lectures on the Diseases of the Urinary Organs.* By Sir Benjamin C. Brodie, Bart., V.P.R.S., Serjeant-Surgeon to the King, and Surgeon to St. George's Hospital. *Second Edition.* —London, 1835. 8vo. pp. 329.

The impulse which every branch of knowledge has of late years received, has been strongly felt by those sciences which have immediate relation to our profession; and each, in generous rivalry, strives to outstrip the others in the rapidity of its progress. Chemistry can boast of her Davy, who, armed with the voltaic battery, has unravelled some of nature's most artful handiwork; and of a Faraday, who at one time compresses aeriform matter into liquid, and the liquid into a solid form,—at another, lifts the veil from magnetic attraction, and gives us at least a glimpse of the secret of its origin. The microscope not only discloses new facts in anatomy, but seems about to reveal the very essence of morbid structure; Vegetable becomes the rival of Animal Physiology; while Medical Jurisprudence, rising into the rank of a science, points to detected murderers (like serpents strangled in its cradle,) and gives promise of the most gigantic labours. Physic has pressed the ear into her service, and, by means of the stethoscope, can watch the progress of disease in the living body, with a hitherto unattainable accuracy. Amidst all these competitors, Surgery need not hold down her head, but, appealing to the splendid discovery of Elderton, Civiale, and Heurteloup,—to the operative dexterity of an Abernethy or a Mott,—or to the more laborious investigations of Cooper and Brodie, who, dividing between themselves the frame of man, have travelled through the whole province of chirurgical disease, marking out the boundaries that divide cognate maladies,—may not merely claim an equality with her rivals, but assert her superiority in the brilliancy and utility of her achievements.

It is the peculiar characteristic of the recent improvements in our profession, that they are chiefly of a practical nature; whereas, in the last age of the art, (if we may be allowed to make a division in what is essentially continuous,) its principles were more particularly investigated. It is true that Pott and Hey, Desault and Richter, must be reckoned among the greatest practical surgeons; but the labours of the Hunters and Bichat so much surpass all others, that they may with justice be adduced as stamping the character of the times in which they lived. But, in the present day, the distinguished surgeons of this country, whom we have before named, with Dupuytren, Scarpa, and others, abroad, have employed themselves in the analysis of disease, rather than in abstract researches into the nature of morbid action. Like the cultivators of natural history, they have often shown that the genera established by their predecessors were too comprehensive, and that the species were in fact frequently generically distinct; and, discarding the nomenclature derived from some prominent peculiarity or fanciful hypothesis, they have substituted one founded on the morbid alterations of structure. Thus, the name of white swelling is no longer employed to designate every variety of diseased joint; but inflammation of the synovial membrane, ulceration of the cartilage, &c. are now separately recognised; and the term spina ventosa is obsolete,—necrosis, abscess of the bone, &c. being accurately understood and identified.

We may go further, and assert that many. of our recent improvements consist of the practical application of principles investigated in the last age. For, if to Hunter we owe any accurate idea of the nature of union by the first intention, Sir Astley Cooper has the credit of having taught that lint, dipped in the blood of the patient, forms the best cement for a recent breach of surface, and has employed it with such success, that lacerated wounds of joints, and compound dislocations, are now not unfrequently saved from amputation. The great surgical physiologist of the last century first conceived the plan of arresting the course of the blood between the heart and an aneurismal tumour; but surgeons of our own times have thrown the ligature around the subclavian, innominata, and iliac arteries. William Hunter and Bichat explained the structure and functions of the synovial membranes; and Sir Benjamin Brodie has followed in their wake, describing the various diseases by which they may be affected. Other instances might easily be adduced, but we shall leave them to the ingenuity of the reader: we have said enough to prove the rapid advance of the art of surgery, more especially in its practical department.

We would not, however, be understood to say that the present age is so selfish in its labours as to reap the harvest of its predecessors' discoveries, without sowing other seed for the benefit of its successors. Messrs. Bell, Mayo, and Magendie have laid open an extensive field, the cultivation of which has been already commenced by Mr. Swan, but in which there is still room for other labourers. Dr. Prout also has thrown so much light on the chemical changes of the secretions of the kidneys in the different diseases of the urinary organs, that great improvements in their treatment might naturally have been anticipated.

It may be thought that in these observations we have spoken rather of the art than of the author before us; but our answer to such a charge would be, that, in reviewing the progress of the one, we in reality follow only the steps of the other. The advance of such men as Sir Benjamin Brodie constitutes the progress of the art in which they labour; for the point at which they halt forms for the time its limits. Those of our readers, therefore, who are anxious to keep in the front rank of their profession, will excuse our introducing an article on the few additions to be found in the second edition of this work; while our seeking the pretext of their existenc eagain to study his opinions, is the highest, no less than the justest, tribute of praise that we can offer to the author.

These additions arise from Sir Benjamin Brodie's recent experience, which has enabled him to acquire further information, both on the history and treatment of some of these diseases. We propose to consider each of these alterations consecutively as they occur in the work, omitting all that is to be found in the former edition; and we must therefore beg the indulgence of our readers, if our observations appear to want those natural links which should connect them together.

The proposition of dividing strictures of the urethra with a cutting instrument, introduced along the canal, originating, we believe, with M. Amussat, but brought into notice in this country by Mr. Stafford, has of late years attracted much attention. We scarcely do justice to Mr. Stafford, in only attributing to him the introduction of the plan into this country; for the instrument which he uses is so decidedly superior to that employed in France, that he deserves to share the credit of the inventor. Sir Benjamin Brodie does not speak very warmly in its praise: the following are his observations upon it.

"Cases may perhaps occur in which this method may be useful, but they are undoubtedly very few in number, and great caution will be necessary on the part of the surgeon whenever he ventures to have recourse to it. If it be required at all, it can only be in cases of very old stricture. But an old stricture occupies a considerable portion of the urethra, and in the division of it there must be always danger of the cutting instrument penetrating into the surrounding cellular membrane; an accident which may be followed in some instances by extravasation of the urine, and the death of the patient; and in others by the formation of a new passage, either below or by the side of the old one, with all its attendant inconveniences, instead of the restoration of the old one." (P. 57.)

Our author's objections seem, however, to touch merely the abuse of these instruments: he points out the danger which want of skill may occasion, but he does not meet the broad question of what profit may be derived from this instrument in dexterous hands. This may perhaps arise from the consciousness of the importance which would be attached to his opinion by the profession; but, as we are not similarly fettered, we shall venture to make a few remarks on the intrinsic merits, both of the plan and instruments.

It is obvious that Mr. Stafford's plan is excellent in theory, for the most strenuous objectors to his instrument will not deny that the process of cutting the stricture, which may be done in a few minutes, is much more summary than that of dilatation, which requires many weeks, if not months, for its accomplishment. They may perhaps affirm, that the stricture in such cases is more likely to return, inasmuch as a cicatrix has always a disposition to contract. This may or may not be: every stricture will return, if a bougie be not occasionally passed; and we cannot consider it any serious objection to this plan, that the bougie must be employed every fortnight, instead of every month, since its passage is attended with no inconvenience. But here, as in other cases, practice has followed theory with a limping step. Mr. Stafford's instruments are either straight, or they have the common urethral curve; the former being intended for the whole anterior part of the canal, the latter for such obstructions as occur after it turns upwards to open into the bladder. The form of these instruments depends upon the supposed course of the urethra, which is said to be straight for the greater part of its length. It is here that the difference of our opinion from the inventor's begins; for, though a straight instrument will readily pass along this portion of the canal, the urethra is not naturally straight. A portion of it is tied up to the pubis by the triangular ligament, while the part immediately in front of it is drawn downwards and backwards by the sphincter ani and transversi muscles. It is said that elongation of the penis will draw the canal into a straight line: this may be true in the dead body, but we doubt its truth in the living. The sphincter ani, in nine cases out of ten, contracts powerfully on any attempt to pass a bougie, and must render the course of the urethra somewhat irregular; and any, the slightest, irregularity renders the use of this instrument dangerous. But there is another objection of equal importance. The thickening of the parietes of the urethra, which constitutes the stricture, does not take place equally in every direction; the lower part being generally more affected than the upper. Now, these instruments, cutting on each side, are as likely to wound the sound as the diseased parts; and hence may follow the train of evils mentioned by Sir Benjamin Brodie. If such are the dangers of the straight instrument, we need not say that they are multiplied when it is curved.

We have here stated its inherent faults; but there is a still stronger objection to its general employment. It is extremely difficult to distinguish many cases of spasmodic from permanent strictures, and instances have come to our own knowledge where the operation has been proposed by persons perfectly competent to use the instrument, but where it was afterwards proved that no permanent stricture existed. Of course, in such cases it is more than dangerous.

No surgeon can have been long in practice without having been consulted on cases of the vascular tumour of the meatus urinarius of the female, first described by Sir Charles Clarke; and none who have met with this disease but will be glad to acquire any additional information as to the best method of effecting its permanent removal. Our author has always recommended, after its excision, the use of the potassa fusa; but this plan was generally followed, in spite of every care, by excoriation of the adjoining parts. In order to prevent this, he has invented a shield, consisting of "a silver tube, incomplete in one part of its circumference, so that, when introduced into the urethra, it allows the caustic to be applied to the tumour, while the sound part of the urethra is defended from it." (P. 72.)

We were surprised that, in the former edition of this work, no mention was made of injections as a means of cure in inflammation of the bladder; more especially as we remembered that, in the lectures of which this book is the corrected transcript, very valuable instructions were given as to the method of employing them, and the benefits to be expected from them. It now appears that this silence was occasioned by our author's well-known caution in promulgating his opinions, until their truth has been tested by long experience. The deficiency is supplied in the present edition.

"The bladder is accessible to local applications, and the question will here arise, 'Can nothing be done for the patient by means of remedies of this description?' The following are the results of my experience on this subject.

"In aggravated cases of the disease, where the symptoms are at their greatest height, the mildest injections, even those of tepid water, will do harm rather than good. They are especially to be avoided where the mucus deposited by the urine is highly tinged with blood. When, however, the symptoms have in some degree abated, the injection of tepid water or decoction of poppies is in many instances productive of excellent effects. An elastic gum catheter may be introduced into the bladder, and the injection may be made by means of a small elastic gum syringe. The liquid should be allowed to remain in the bladder about thirty or forty seconds, and not more than an ounce and a half, or two ounces, should be injected at each time. If the bladder be distended, so as to occasion any considerable degree of pain, the effect is always injurious instead of being beneficial. This operation may be repeated, according to circumstances, once or twice in twenty-four hours.

"When there is a further abatement of the symptoms, the disease having assumed a still more chronic form, and the mucus being free (except on extraordinary occasions) from all admixture of blood, we may venture to add to the injection a very small quantity of nitric acid. At first the proportion ought to be not more than that of one minim of the concentrated, or ten minims of the diluted nitric acid, to two ounces of distilled water; but afterwards this proportion may be doubled. I do not say that it should never be increased still further, but I have observed, that for the most part injections, which are stronger than this, are not only not useful but actually prejudicial. In having recourse to this mode of treatment, it is better to wash out the bladder first with a little tepid water; then to inject the acid solution, allowing it to remain not more than thirty seconds in the bladder. At first the operation should not be repeated oftener than once in every two days; afterwards it may be repeated once daily, but never more frequently than this. If the urine drawn off by the catheter be tinged with blood, the injection should be deferred to the following day; and if the injection be at any time followed by pain, and other symptoms indicating an increase of inflammation, it ought not to be had recourse to again until these have subsided.

"I was first led to adopt the use of the injections of nitric acid in the year 1826; and from the experience which I have now had of them, I do not hesitate to say, that, if the precautions, which I have suggested, be properly observed, they will be found to form a valuable addition to our stock of remedies to be employed in these cases. They are useful not only where the chronic inflammation is the primary disease, but also where it occurs as a secondary affection, the result of a calculus in the bladder, or a chronic enlargement of the prostate gland." (P. 91.)

Every surgeon who has been in the habit of using these injections will coincide with Sir B. Brodie in his opinion of their extreme utility, though each may differ somewhat in the details of the method to be employed.

There is one point, namely, the ill effects of distending the bladder, on which our experience has led, or perhaps seduced us, to a different conclusion from our author. Sudden and violent distention of the bladder is no doubt prejudicial, and has a tendency to increase, rather than diminish, the inflammation; but we have found that a gradual distention, so far from being injurious, is highly beneficial, by diminishing its irritability, and removing some of its most distressing symptoms. The necessary instruments are a catheter, similar to that used in the operation of lithotrity, (but with an extremely small opening, so as to secure the gradual entrance of the fluid into the bladder,) and its appropriate syringe. The following case will exemplify our method of employing the injection, and the success attending its use.

Mr. W. K., ætat. fifty-four, a tradesman residing at Camden Town, placed himself under our care, for what was supposed to be stone in the bladder, on the 12th of February, 1835. He had suffered more or less from his present symptoms about three years, and had tried a variety of remedies, without any good effect. His complaints were, a very frequent desire to pass water, which awoke him seven or eight times every night, (the quantity of urine seldom exceeding an ounce at one time;) pain both before and after its evacuation, which was accompanied with violent straining; the urine was alkaline, of a brown colour, dreadfully fetid, and deposited a large quantity of ropy adhesive mucus, mixed with phosphate of lime, and occasionally streaked with blood; the bowels were very irritable, acting almost every time that he passed water; the countenance was pale, and the whole frame emaciated. On introducing a silver catheter, no obstruction was found in the urethra; but the third lobe of the prostate gland was slightly enlarged, so that about an ounce of urine was retained in the bladder, after the patient had endeavoured to empty it. No stone was discovered by the sound; but the bladder was so contracted, that the injection of two ounces of warm water occasioned very violent pain. He was ordered, on his return home, to use an enema, containing thirty minims of tincture of opium.

On the 15th, he expressed himself much relieved by the injection, the pain being the symptom most alleviated. Four ounces of warm water were introduced, but the last ounce and a half caused very severe pain. The whole quantity was not injected at once, but only sufficient at first to distend the bladder fully. The stopcock was then turned, till the patient said he had no desire to pass water. Half an ounce or more was then admitted into the bladder: this caused considerable pain; but, on again turning the stopcock, so as to prevent the entrance of more fluid, the pain subsided in three or four minutes. The remainder was then injected, but the pain produced was so violent, that the whole was immediately drawn off.

On the 18th, he reported himself much better. He awoke only three times during the previous night to pass water, and the quantity passed was nearly two ounces each time. The injection was repeated, but six ounces of fluid were introduced; the quantity being guided by the resistance to the descent of the piston of the syringe, and the degree of pain felt by the patient.

21st. Better. Nearly eight ounces of warm water injected.

25th. He calls himself quite well, but the bladder does not completely empty itself, though it can bear a tolerable quantity of urine. Ordered to introduce a flexible catheter for himself daily. Rather more than half a pint injected.

March 15th. He bore today thirteen ounces of fluid in the bladder. The urine is no longer alkaline; the mucus has disappeared, excepting small flocculi that float in the water. He has no pain; the bowels are comfortably open; and in appearance he is altogether an altered man.

April 1st. No larger quantity of water than the thirteen ounces has been injected, but it now causes no pain; and, as he has no unpleasant symptoms remaining, he is recommended to introduce a flexible catheter once a week, after passing water, in order to ensure the bladder being emptied, and to discontinue his attendance.

It is this case, with others which we could mention, that induces us to differ from our author, as to the pernicious effects of violently distending the bladder in cases of chronic inflammation. We have no doubt that instances do occur where it may be hurtful, but we do doubt whether, in these, the inflammation does not partake of an acute character; in which case, any violence is likely to be followed by dangerous symptoms.

We now arrive at the most important addition which the work has received. In a note appended to the last edition, the author regretted his inability to complete the subject of the diseases of the urinary organs, by introducing a systematic notice of those affecting the kidneys. We have in the present edition, not indeed a systematic notice, but some first steps towards an account of these diseases. We shall, without preface, abstract the author's account of the symptoms affecting the bladder, in consequence of disease of the kidney.

"Calculi of the kidney occasionally produce symptoms, which are referred to the bladder rather than to the kidney. I shall have occasion, in a future Lecture, to notice a well marked example of this fact, which occurred in my own practice; and you will find others referred to by Morgagni. 'A patient,' says this eminent pathologist, 'complained of very little pain in the region of the kidney; .while he was tormented with pain in the bladder so excruciating, that five or six physicians who attended him entertained no doubt that the seat of the disease was in that organ. On dissection, however, no morbid appearance whatever was discovered in the bladder, but there were large and ramifying calculi of the kidney.'

"If calculi of the kidney produce symptoms which may easily be mistaken for those of disease in the bladder, it may reasonably be expected that some other diseases of the kidney should affect the bladder in the same manner. Several years have elapsed since I was first led to suspect this to be the case, and the result of all the experience which I have since had has been to remove whatever doubts I might formerly have entertained on the subject. Whoever is much engaged in this branch of surgical practice will meet with a number of facts, which cannot so well be explained on any other hypothesis, and which collectively form such a mass of circumstantial evidence, as is almost irresistible, in favour of the opinion 'that the worst symptoms of irritable bladder may occur as a consequence of chronic inflammation, abscess, or other organic disease of the kidney, the bladder itself, and the organs in immediate connexion with it, having been free from disease in the first instance.'

"The opportunities of obtaining direct or positive evidence (that is, by means of post-mortem examination), on a point like this, are of comparatively rare occurrence; for so intimate is the union of the different organs which constitute the urinary system with each other, that disease can scarcely exist for a great length of time in one of them, without extending in a greater or less degree to the rest. Such opportunities are, however, occasionally met with where the patient has died before the disease has reached its most advanced stage; and I am able to adduce the following interesting history in illustration of the foregoing observations.

"A gentleman consulted me in November, 1833, labouring under the following symptoms: He voided his urine frequently, and in quantities varying from an ounce to an ounce and a half. Always after making water he had a severe pain lasting a few minutes, and extending along the course of the urethra. The urine was pale, semi-opaque, of an acid quality, and, when tested with heat and nitric acid, it was found to be highly albuminous. Occasionally, small masses of a substance resembling coagulated albumen were seen floating in it. He made no complaint of pain in the loins; he was able to empty his bladder by his own efforts, and the urethra was free from stricture. There was no calculus in the bladder, nor had sand or gravel ever been observed in the urine. These symptoms had begun to exist in the preceding February, since which time they had gradually increased. For a short time during the month of March, the urine had been tinged with blood.

"In addition to these local ailments, the general health was much impaired: the patient had lost flesh, was languid, dejected, and of a pallid countenance.

"Soon after I was consulted, the urine became again tinged with blood. The bodily powers continued to fail, and the local symptoms became more urgent. There was a total loss of inclination for food, the extremities became cold, the pulse feeble, and he died at the end of February, 1834.

"On examining the body after death, the kidneys were found to be of a dark colour from excessive vascularity, and of a soft and somewhat brittle consistence; the distinction between the cortical and tubular positions being less marked than under ordinary circumstances. The investing membrane of the kidney had a very slight adhesion to the kidney itself, but it adhered very closely to the adipose substance of the loins. On the surface of each kidney, and partly imbedded on its substance, were four or five membranous cysts, each of the size of a large pea: and in one of them there was a similar cyst, but as large as a nutmeg, completely imbedded in the cortical substance. The pelvis, infundibula, and ureters, were not more capacious than under ordinary circumstances; but, on their being slit open, their internal membranous surface presented the appearances of considerable inflammation.

"It could not be said that the bladder was found altogether free from disease, but the morbid appearances were so slight, compared with those observed in the kidney, that it seemed impossible to doubt that the last-mentioned organ had been the seat of the primary disease, and that the latter was affected only in a secondary manner. It was contracted, and the muscular tunic was somewhat thickened; but not more so than they must have been in a person, who from any cause had been teased for a considerable time by an incessant inclination to void his urine. The vessels of the mucous membrane were turgid with blood, but not in the same degree as those of the membranous parts of the kidneys.

"When we say that there is an irritable state of the bladder, depending on disease of the kidney, it is of course intended to express that the inclination to void the urine occurs at short intervals, and when there is but little urine in the bladder. In addition to this there is sometimes a cutting pain, either confined to the neck of the bladder, or extending forward in the course of the urethra after each effort to make water; at other times there is no pain whatever, beyond that which is always felt when the bladder is distended with more urine than it can well contain. In most instances there is some degree of pain in the loins, but in some there is none whatever. The urine is generally albuminous, being voided slightly turbid, and becoming opaque on the addition of nitric acid; or, if not albuminous constantly, it is so occasionally. In some instances it is voided of the colour of whey, and, on being allowed to stand, deposits a purulent sediment. It is generally acid, and occasionally tinged with blood. Small globular masses of semitransparent lymph are sometimes seen floating in it; and in one instance the patient used to void, at intervals, considerable masses of opaque, laminated, firmly coagulated lymph, of an oblong and somewhat conical shape. These seemed to have been moulded in their passage down the urethra, and, before they came away, they produced symptoms similar to those which precede the expulsion of renal calculi." (P. 95.)

Such is the affection of the bladder, according to the author, sympathetic with disease of the kidney; but he adds a caution, not to value these symptoms at more than they are worth, as it is his opinion that future investigations will throw more light on the subject.

The treatment which Sir B. Brodie has found most efficacious is as follows:

"In the treatment of these cases you will find that opiates, whether exhibited by the mouth or in the form of clyster, are productive of a temporary advantage; and these remedies may be employed on special occasions, when the symptoms are more than commonly distressing. There are, however, well-founded objections to the constant use of opiates, inasmuch as they do nothing more than palliate the symptoms, without removing the cause which produces them; and as they are always liable, after a certain time, to injure the patient's general health, by confining the bowels, and deranging the secretions of the liver. A gentleman whom I attended formerly, suffering from a most severe irritation of the bladder, in consequence of disease in the kidneys, discovered another and a more innocent method of giving himself relief. Once daily he introduced an elastic gum catheter, through which he injected into the bladder as much tepid water as it was capable of containing. At first, the bladder would admit only a very small quantity, perhaps an ounce; but by degrees he was able to inject a larger quantity, amounting at last to half a pint or more; and now, instead of voiding it every twenty or thirty minutes, he was able to retain his urine for two hours or even longer. Thus he contrived to keep himself in a state of comparative comfort; but the relief was only temporary, and as soon as he omitted the use of the injection, the bladder relapsed into its former habits.

"It is reasonable to suppose, that the patient will profit most from the use of those remedies which are directed to the original seat of the disease; blisters, setons, and issues in the loins, may be had recourse to, and are productive of the best effects. Their influence over the disease is most marked in those cases, in which there are deposits of pus, or small masses of coagulated lymph, floating in the urine; the former becoming in some instances very much diminished, and the latter even disappearing altogether, soon after the counter-irritation has been well established. The *uva ursi* has had a doubtful reputation as a remedy in cases of disease of the bladder, some believing it to be of great efficacy, and others attributing to it no efficacy whatever. My own experience would lead me to suspect that its influence is confined to the cases of which I am now treating, but that in these it may in some instances be employed with very great advantage. It must be administered, however, in larger doses than those which I find to be in common use. Thus from ʒj. to ʒij. of the extract may be given in pills daily, or Oss. to Oj. of the following infusion, which has appeared to me to be more efficient than the extract:*

℞. Foliorum Uvæ Ursi, ℥j.
Aquæ distillatæ ferventis, ℥xviij. Macera per horas ij., dein decoque ad Oj. et cola.*

"In some instances it has appeared to me that the patient has derived marked benefit from the exhibition of the Tinctura Ferri Muriatis, in doses varying from m.x. to m.xv. three times daily; and, in others, from that of the infusion of wild carrot-seed, given in the following manner:

℞. Seminum Dauci contusorum, ℥j.
Aquæ ferventis, Oj. Macera per horas ij. vel iij. et cola.

The whole of this quantity may be taken daily. None of these remedies, however, produce an immediate improvement, and the patient must make up his mind to persevere in the use of them for a considerable time." (P. 100.)

Our author says nothing concerning the diet to be observed in these cases; but we believe we may add, that a low diet is not attended with the advantage which might be expected, from the antiphlogistic nature of the remedies. Light but nutritious food, in moderate quantities, with even a small quantity of wine, will sometimes be found to calm the irritability of the bladder. We have known an instance of a gentleman suffering under these symptoms, who, in the morning, was incessantly attempting to pass water; but, after dinner, when he had taken two or three glasses of sherry, he would remain undisturbed for several hours.

Sir Benjamin Brodie next propounds two questions, to be

* "When the Uva ursi is taken in large quantity, it imparts a greenish colour to the urine; which, however, is most distinct when that fluid happens to be alkaline; and it may, perhaps, be admitted as a question, whether the good effects which it produces do not immediately arise from the astringent principle which it contains being brought into contact with every part of the kidney, while it is, as it were, being filtered through the minute vessels which belong to its secreting structure."

answered by future investigators. The first is, "Wherefore does it happen that calculi of the kidney, or chronic inflammation or abscess of that organ, should occasion symptoms, such as have been described, in some instances, and not in others?" (P. 103.) As the importance of this query to a scientific line of practice is evident, even a feeble attempt to solve it may be pardoned: we will therefore take the liberty of relating a case, which may at first appear foreign to the subject, but which appears to throw some light upon the point mooted.

Mr. N., a clerk in a public office, was recommended to our care by one of the most eminent physicians in London, in order to ascertain whether he had a stone in the bladder. He complained of severe pain in the loins, which had existed three or four months, and for which he had been cupped. This pain shot down in the direction of the ureters, and affected both the pubes and testicles. After making water, which he did very frequently, he had pain shooting along the canal of the urethra. The urine was pale, and the addition of nitric acid caused the coagulation of a considerable quantity of albumen. He had grown extremely thin, and there was an expression of extreme anxiety in his countenance. He had never passed either sand, gravel, or blood, in his urine. We examined the bladder with a sound, but no stone was discovered, and, excepting that it was somewhat tender at the neck, there appeared to be no disease there. Such were the symptoms as they appeared at the time, and, under the supposition that there was disease of the kidney, he was directed to take small doses of opium, until this opinion could be represented to the physician who had committed him to our charge. He was requested to attend us in three days' time; but on that morning his servant came in his stead, to inform us that, as he was quitting the house for the purpose, he had dropped down dead. On June 5th, 1835, (two days afterwards,) we examined the body. The neck of the bladder was a little more vascular than usual, as also were the kidneys, but hardly enough to constitute disease. The cause of the symptoms was found to be a large aneurism of the aorta, just as it passed through the diaphragm, which had burst into the right pleura. Could its pressure upon branches of the same plexus of nerves as supplied the kidney occasion the similarity of the symptoms?

The second question propounded by our author is of more easy solution.

"What is the effect of long-continued irritation of the bladder, depending on the sympathy of that organ with disease in the kidney, or of the bladder itself? It is reasonable to expect, and it corresponds with what happens in some other analogous cases, that the bladder, thus sympathetically affected, should at last become itself the seat of actual disease. I have related a case, in which, although it was plain that the principal and primary disease was in the kidney, there was also a slight degree of inflammation of the mucous membrane of the bladder. Now, if the patient had lived some time longer, is it not probable that this would have gone on increasing, until the urine had become loaded with a thick, tenacious, alkaline mucus; the patient suffering, at the same time, from those other distressing symptoms with which that morbid secretion is usually accompanied. Other cases have fallen under my observation, in which chronic inflammation of the mucous membrane of the bladder existed to a considerable extent, in combination with disease of the kidney, and in which, from the history of the case, it could not be doubted that the former was altogether a secondary disease; the kidney having been the part primarily affected. As the kidney sooner or later participates in disease which has had its origin in the bladder, so does the bladder ultimately suffer in consequence of disease which began in the kidney." (P. 103.)

A section has been added, in the present edition, on Scirrhus of the Prostate Gland. It will be seen, from our author's expressions, that he has not quite satisfied his own mind of the existence of such a disease; but we leave him to make his own statement.

"I have observed that malignant diseases of the prostate are of rare occurrence. I have, however, seen cases in which I could not well doubt that the prostate was affected by scirrhus, although I had no opportunity of positively ascertaining the fact by dissection.

"In February, 1831, a gentleman from Maidstone, fifty years of age, consulted me under the following circumstances:

"He complained of a too frequent indication to void his urine; so that he was disturbed not less than ten or twelve times in the course of the night. The act of voiding it was attended with some degree of difficulty; and occasionally he observed that it could not be accomplished in the position in which he then was, and he was under the necessity of altering it. A small quantity of urine sometimes flowed involuntarily after he thought that his bladder was emptied. He suffered a severe and constant pain, extending from the left groin across the lower part of the abdomen above the pubes, and also down the thigh and leg. The pain above the pubes was aggravated during the effort to make water. He compared the pain in the thigh and leg to that which he suffered when he had sprained his thumb, and said that it prevented his throwing the weight of his body on that limb when he stood erect. There was an enlarged and indurated gland in the groin, to which the pain was referred. The urine was high coloured, but otherwise in a healthy state, free from mucus and albumen. A catheter having been introduced after he made water, it was ascertained that there was no obstruction in the urethra, and no residuary urine was found in the bladder. He had lost flesh, and had the aspect of a person labouring under a malignant disease. He was hot and feverish at night, and the pulse was never less than one hundred in a minute. The prostate gland being examined from the rectum, was found not very much enlarged, but of a stony hardness.

"The symptoms which I have described first showed themselves in the preceding August, and had gradually increased up to the time of my being consulted.

"It appeared to me that the circumstances of this case were not well to be explained, except on the supposition of the prostate gland being affected with the true scirrhous disease; and Mr. Travers, who was consulted, also came to the same conclusion.

"The patient returned to Maidstone, and died shortly afterwards. There was no opportunity of examining the body after death.

"Another case fell under my observation, in which, also, I was led to believe that the patient laboured under scirrhus of the prostate. I preserved no notes at the time, but I know the following history to be accurate as far as it goes.

"A gentleman, about sixty years of age, who had been long in India, consulted me a few years ago respecting what appeared to be a chronic enlargement of the prostate gland. There was nothing unusual in his symptoms, and I merely recommended him the regular use of the catheter. From this treatment he derived much benefit, and he persevered in it ever afterwards.

"It was not less than five or six years after this period that I was requested to see him again, in consultation with Dr. Latham and Mr. Mawdslay. He now could void no urine without the assistance of the catheter. There was a constant and most severe pain, referred to the neck of the bladder, which was not relieved on the urine being drawn off. The urine deposited a considerable quantity of adhesive mucus, and was of an ammoniacal odour. The prostate gland, examined by the rectum, was found to be much enlarged, and of a stony hardness. From these circumstances we were led to suspect that the prostate had become the seat of a true scirrhous disease; and, in confirmation of this opinion, we found the patient complaining of excruciating pains in various parts of the body, sometimes in one part, sometimes in another, which could be compared to nothing except the pains under which persons afflicted with carcinoma occasionally labour. Altogether, I may say that I have never seen a human being whose sufferings were more intense; and they were scarcely mitigated by the exhibition of very large doses of opium. I continued to visit him occasionally, in consultation, for nearly a year, at the end of which time he suddenly lost the use of the muscles of his lower limbs, and died in a fortnight afterwards. Permission was not obtained to examine the body; but it is worthy of notice, that a lady, whose case is related in the eighth chapter of the last edition of my work on Diseases of the Joints, and who had long laboured under carcinoma of the breast, died after a similar attack of paralysis of the lower limbs, and that in her it was ascertained by dissection, that the cause of the paralysis was a conversion of the bones of the spine into the scirrhous stricture." (P. 161.)

The remaining observations introduced into the present edition have reference to the different methods of extracting a stone from the bladder through the urethra. The following directions for the use of Sir Astley Cooper's urethra forceps, as improved by Mr. Weiss, are so excellent, that we need no apology for quoting them.

"In using these forceps, you should select a pair of as large a size as the urethra will readily admit. If you have reason to believe that the stone is of a very small size, or that there are several small stones, it is better that the opposite surfaces of the blades should be made concave: otherwise, they may be nearly flat, and somewhat serrated. The patient should be laid on his back; and it is generally better that his pelvis should be supported by a thick cushion, so that it may be higher than his shoulders. The first step of the operation is to introduce a silver catheter, and thus empty the bladder of its contents. From five to six ounces of tepid water are then to be injected into the bladder, so as to distend it moderately. If any considerable portion of the water should escape, the injection should be repeated; *it being absolutely necessary that the operation should never be attempted on an empty bladder*. The forceps are next to be introduced, and of course in their closed state. They are first to be used as a sound, so as to ascertain the exact situation of the calculus. If this be not readily detected, the forceps may be withdrawn, and an ordinary sound may be used instead: or the patient may be directed to turn on one side, placing himself on his back again afterwards; by which change of position the stone may probably be made to roll into some more convenient place, so as to be more within reach of the forceps. The forceps are then to be cautiously opened over the stone, and afterwards closed upon it. By this simple management, with a light hand, the stone is seized with facility, in the great majority of cases: otherwise, you may succeed by the following method, which is especially adapted to those cases in which the bladder contains several stones of a very small size.—Let the forceps be opened with the convexity of its blades pressed against that part of the bladder which is towards the rectum, so as to make it the lowest or most depending situation. Then, by a slight motion given to the handle of the instrument, the stones are made to roll into its grasp; and thus I have sometimes been enabled to remove several small stones at once.

"The advantage arising from the elevation of the pelvis is, that the stone is then less liable to be lodged near the neck of the bladder, where the seizing it is always more difficult than when it lies near the fundus. Attention to this point is especially of importance in cases of enlarged prostate. Sometimes, however, when the stone is very large, notwithstanding this precaution, it will remain in the hollow behind the prostate. In order to seize it, you must then turn the forceps round, so that the convex part of it may be towards the pubes, and the extremity towards the rectum. This part of the operation, however, must be executed with great caution, as, otherwise, serious injury might be inflicted on the prostate, or even on the bladder itself.

"When the stone is grasped, you may know exactly its diameter by means of a scale fixed to the handle of the forceps. If it be of a very small size, you have only to withdraw the forceps from the bladder in the usual manner, and the stone with it. If it be of a very large size, so that it is evident that it cannot be made to enter the urethra, you need only open the forceps again to set the stone at liberty ; and you may then determine, at your leisure, what other method should be adopted for the patient's relief. But the forceps may seize a stone of an intermediate size; one which may be made to enter the urethra to a certain distance, being then stopped by some narrow portion of the canal. The neck of the bladder is very easily dilated, and a stone of considerable size may be drawn into that portion of the canal which lies in the perineum. It may then be very distinctly felt through the integuments behind the scrotum, and if a small incision be made on it in this situation, it is easily extracted; the forceps, after the removal of the stone, being closed, and withdrawn in the usual manner. I have performed this operation several times, and have extracted stones of more than an inch in one, and of nearly an inch in another, diameter. It is so simple, that, in two instances in which I had recourse to it, although I had no pair of hands to assist me but my own, it was not attended with the smallest difficulty. The patient should be directed to remain in bed afterwards, and an elastic gum catheter should be allowed to remain in the urethra and bladder, for the purpose of drawing off the urine, and preventing it dribbling through the wound. With this precaution, the wound will, in some instances, be enabled to heal in less than a fortnight.

"But it will sometimes happen, that a stone which is easily drawn through that part of the urethra which lies in the perineum, meets with an impediment in the anterior part of the canal; that is, either at the external orifice, or exactly at the anterior part of the scrotum, or somewhere in the intermediate space. If the impediment be close to the orifice, that part is easily dilated by means of a probe-pointed bistoury, and if it be in some other part of the canal, you may remove it by means of an incision made through the skin, *corpus spongiosum*, and membrane of the urethra. Let me caution you, however, never to make such an incision into the urethra close to the scrotum. It is difficult, even by the constant retention of an elastic gum catheter, to prevent a small quantity of urine finding its way into the loose cellular texture of the scrotum; and this may be productive of a succession of troublesome abscesses, or even of dangerous consequences. Such experience as I have had, would make me altogether more averse to the excision of stones from the urethra, where it is connected with the penis, than where it lies in the perineum; and a very simple contrivance makes such a proceeding altogether unnecessary. The forceps should be constructed so as to admit of the introduction of a screw, by means of which considerable pressure may be made on the moveable blade. When the stone is felt through the *corpus spongiosum*, and can be drawn no farther without lacerating the membrane of the urethra, instead of using the knife, introduce the screw, and it is immediately crushed: after which the difficulty of extracting it is at an end. If a few fragments remain in the urethra, they are forced out the next time that the patient has occasion to void his urine." (P. 245.)

It is of the utmost importance that surgeons, who intend to use this instrument, should practise with it frequently on the dead body; or what is both a safe and easy operation, becomes difficult, tedious, and dangerous. We have seen the most severe inflammation of the bladder follow long and ineffectual efforts to extract a stone, while others have been relieved in less than a minute of all their sufferings. We are not advocates for operating in general by the stopwatch, and we scarcely admire the man who can lithotomize in a minute; but in this operation celerity is safety, and the only danger arises from its prolongation.

The last portion of the work which we shall extract is our author's comparison between the merits of lithotrity and lithotomy. After giving a slight sketch of the history of the former, (in which he gives the credit of the original idea to General Martin and Mr. Elderton,) he thus proceeds:

"It must be acknowledged that this operation possesses several advantages over lithotomy. It is less formidable to the patient. It requires little or no confinement; and many individuals will be induced to submit to it at an early period, who would not have mustered courage to submit to lithotomy, until after a lapse of time, when their sufferings had become excessive, and when, probably, circumstances had arisen to render the operation dangerous. To this may be added, that there is no danger of hemorrhage, nor of those ill consequences which arise from an incision or laceration extending into the loose cellular structure which surrounds the neck of the bladder.

"But it is scarcely to be expected that any method can be devised of relieving human nature of so great a calamity as that of a stone in the bladder, which shall be altogether free from difficulty and danger; and it is no disparagement to so great a discovery, as that of the lithontriptic operation, to enumerate the following as the principal disadvantages belonging to it. The patient does not obtain a cure at once; and in many instances, the process, by which the stone is crushed, requires to be repeated several times. As the smallest fragment which remains behind will form the nucleus of a new stone, a recurrence of the disease is more likely to take place after the lithontriptic operation, than after that of lithotomy, especially in those cases, in which, in consequence of an enlargement of the prostate gland, the patient is unable completely to empty his bladder. The operation is by no means well adapted, except to calculi of moderate size; and when applied to those of larger dimensions, it is either altogether impracticable, or difficult, tedious, and dangerous. When the stone is large, the sharp angular fragments lying in the bladder induce inflammation of its lining membrane, attended with severe local suffering, producing afterwards much disturbance of the general system, and either retarding the cure, or terminating in the patient's death. It is further to be observed, that those complications of disease in the kidneys, or bladder, or ulcerated prostate, which render the old operation hazardous, render the new operation hazardous also; so that the invention of the latter does not relieve us of those cases, in which experience has taught us to shrink from the performance of the former. As to the quantity of actual suffering which the patient has to undergo under one of these methods, as compared to that which belongs to the other, it is not possible to arrive at any satisfactory conclusion. The fact is, that the amount of pain is, for the most part, over-estimated; and that those who have gone through either of these operations, generally say afterwards, that the pain was less than they had expected. This observation, at least, applies to those cases, in which the bladder is healthy, and the operation proceeds favorably. If the bladder be inflamed, or any thing occurs to render the operation difficult and tedious, the patient undoubtedly suffers for the time severely, whether the stone be crushed, or extracted by incision." (P. 315.)

In conclusion, Sir B. Brodie recommends the employment of Weiss's screw lithotrite, as preferable to the hammer apparatus of Heurteloup; and, from experience, we can coincide in the recommendation.

We now terminate our review, or rather our abstract of the fresh instruction afforded to the profession in this edition. In doing so, while we feel, on the one hand, that praise is here a work of supererogation, and that the unanimous verdict of every competent judge does not require the concurrence, however cordial, of an anonymous reviewer; we are equally sensible that it would be absurd, as well as uncourteous, to conclude without remarking, that the present is one of the few productions of our time, which no practitioner can neglect to study, without injustice to himself and to his patients.

